# Effects of β-alanine supplementation during a 5-week strength training program: a randomized, controlled study

**DOI:** 10.1186/s12970-018-0224-0

**Published:** 2018-04-25

**Authors:** José Luis Maté-Muñoz, Juan H. Lougedo, Manuel V. Garnacho-Castaño, Pablo Veiga-Herreros, María del Carmen Lozano-Estevan, Pablo García-Fernández, Fernando de Jesús, Jesús Guodemar-Pérez, Alejandro F. San Juan, Raúl Domínguez

**Affiliations:** 1 Department of Physical Activity and Sport Sciences, Faculty of Health Sciences, Alfonso X El Sabio University, Avda, Universidad 1, Building C, 3rd floor, Office C-A15, Villanueva de la Cañada, 28691 Madrid, Spain; 2 Department of Physical Activity and Sport Sciences, TecnoCampus, College of Health Sciences, Pompeu Fabra University, Ernest Lluch, 32 (Porta Laietana), 08302 Mataró-Barcelona, Spain; 3 Department of Pharmacy, Faculty of Health Sciences, Alfonso X El Sabio University, Avda, Universidad 1, Building C, 3rd floor, Office C-A04, Villanueva de la Cañada, 28691 Madrid, Spain; 4 Department of Pharmacy, Faculty of Health Sciences, Alfonso X El Sabio University, Avda, Universidad 1, Building D, 3rd floor, Office D-342, Villanueva de la Cañada, 28691 Madrid, Spain; 5 Department of Physiotherapy, Faculty of Health Sciences, Alfonso X El Sabio University, Avda, Universidad, 1, Building C, 3rd floor, Office C-H05, Villanueva de la Cañada, 28691 Madrid, Spain; 6 Department of Pharmacy, Faculty of Health Sciences, Alfonso X El Sabio University, Avda, Universidad 1, Building D, 3rd floor, Office D-348, Villanueva de la Cañada, 28691 Madrid, Spain; 7 Department of Physiotherapy, Faculty of Health Sciences, Camilo José Cela University, Urb, Villafranca del Castillo, Calle Castillo de Alarcón, 49, Villanueva de la Cañada, 28692 Madrid, Spain; 8 Department of Health and Human Performance. Faculty of Physical Activity and Sport Sciences, Polytechnic University, Social Building, 2nd floor, Office 205, Madrid, Spain; 9 Department of Physical Activity and Sport Sciences, Faculty of Health Sciences, Alfonso X El Sabio University, Avda, Universidad 1, Building C, 3rd floor, Office C-A12, Villanueva de la Cañada, 28691 Madrid, Spain

**Keywords:** β-alanine, One-repetition maximum test, Exercise program, Average power, Jump height

## Abstract

**Background:**

β-Alanine (BA) is a non-essential amino acid that has been shown to enhance exercise performance. The purpose of this investigation was to determine if BA supplementation improved the adaptive response to five weeks of a resistance training program.

**Methods:**

Thirty healthy, strength-trained individuals were randomly assigned to the experimental groups placebo (PLA) or BA. Over 5 weeks of strength training, subjects in BA took 6.4 g/day of BA as 8 × 800 mg doses each at least 1.5 h apart. The training program consisted of 3 sessions per week in which three different leg exercises were conducted as a circuit (back squat, barbell step ups and loaded jumping lunges). The program started with 3 sets of 40 s of work per exercise and rest periods between sets of 120 s in the first week. This training volume was then gradually built up to 5 sets of 20 s work/60 s rest in the fifth week. The work load during the program was set by one of the authors according to the individual’s perceived effort the previous week. The variables measured were average velocity, peak velocity, average power, peak power, and load in kg in a back squat, incremental load, one-repetition maximum (1RM) test. In addition, during the rest period, jump ability (jump height and power) was assessed on a force platform. To compare data, a general linear model with repeated measures two-way analysis of variance was used.

**Results:**

Significantly greater training improvements were observed in the BA group versus PLA group (*p* = 0.045) in the variables average power at 1RM (BA: 42.65%, 95% CI, 432.33, 522.52 VS. PLA: 21.07%, 95% CI, 384.77, 482.19) and average power at maximum power output (*p* = 0.037) (BA: 20.17%, 95% CI, 637.82, 751.90 VS. PLA; 10.74%, 95% CI, 628.31, 751.53). The pre- to post training average power gain produced at 1RM in BA could be explained by a greater maximal strength gain, or load lifted at 1RM (*p* = 0.014) (24 kg, 95% CI, 19.45, 28.41 VS. 16 kg, 95% CI, 10.58, 20.25) and in the number of sets executed (*p* = 0.025) in the incremental load test (BA: 2.79 sets, 95% CI, 2.08, 3.49 VS. PLA: 1.58 sets, 95% CI, 0.82, 2.34).

**Conclusions:**

β-Alanine supplementation was effective at increasing power output when lifting loads equivalent to the individual’s maximal strength or when working at maximum power output. The improvement observed at 1RM was explained by a greater load lifted, or strength gain, in response to training in the participants who took this supplement.

**Electronic supplementary material:**

The online version of this article (10.1186/s12970-018-0224-0) contains supplementary material, which is available to authorized users.

## Background

β-Alanine (BA) is a non-essential amino acid synthesized in the liver [1]. It is also found naturally ocurring in animal products such as pork, chicken or red meat [2]. The dietary supplement classification system of the *Australian Institute of Sport* (AIS) describes BA as a class A supplement based on the level of evidence shown for its beneficial effects on sport performance [3].

The effect of BA on performance has been attributed to its capacity to increase carnosine synthesis. Carnosine is a dipeptide composed of the amino acids BA and L-histidine [4]. As the organism is incapable of directly absorbing carnosine [1] and as it known that, unlike L-histidine, BA is able to increase muscular carnosine reserves [5], its ingestion is considered the limiting factor for muscular carnosine synthesis [4, 6]. In effect, the intake of 4.8–6.4 g/day of BA over a period of 5–6 weeks has been noted to increase muscular carnosine concentrations [7, 8].

As the major intracellular buffering protein [9], the main function of carnosine is pH regulation [10]. Carnosine promotes the sensitivity of muscle fibers to calcium [11, 12], enhancing muscle excitation-contraction [11, 13, 14]. These effects have determined that BA supplementation improves performance at exercise efforts of duration 6 to 60 s [15–17]. In these short, high-intensity exercise movements, glycolytic energy metabolism prevails over the high energy phosphagen system and over oxidative phosphorylation [18].

Among the different studies that have examined the effects of BA supplementation, only a few have focused on its impacts on resistance exercises [19, 20]. Thus, Outlaw et al. (2016) [20] found that supplementation gave rise to a larger number of leg press repetitions executed with a load equivalent to 65% of the individual’s one-repetition maximum (1RM). Hoffman et al. (2006) [19] noted that the intake of both BA and creatine improved the load lifted in a 1RM squat test.

Although BA supplementation may help increase the 1RM [19] and the maximum number of repetitions conducted at a submaximal load [20], no study has yet examined the effects of supplementation on power output in resistance training. Power is related to force and velocity. As muscular power production is one of the main determinants of sport performance [21, 22], several studies have assessed the effects of caffeine supplements on power output in resistance exercises such as back squat (BS) [23, 24], detecting an ergogenic effect on power production.

Another important factor to consider in sports training is the quantification of fatigue, defined as an incapacity of the neuromuscular system to maintain a given power level [25]. The countermovement jump (CMJ) is a movement that reflects the contractile and neuromuscular control properties of the whole locomotor system [26]. Thus, monitoring jump height loss during an exercise session has been used as an indicator of muscular fatigue. Several studies have confirmed a loss of CMJ height during various resistance training exercises [27–33]. However, so far no study has monitored CMJ jump height while conducting a 1RM test or the effects of BA supplementation on this indicator of fatigue.

Given the scarce investigations exploring the influence of BA on performance in resistance exercises [19, 20], the aim of the present study was to examine the effects of BA supplementation during a 5-week resistance training program. The primary outcome for the study was power output in a BS incremental load test. Secondary outcomes were kilograms lifted and lifting velocity during the test. As tertiary outcomes, we also examined the jump height and average power losses produced after exercise in a CMJ test. We hypothesized that BA supplementation would improve power output, kilograms lifted and movement velocity during the incremental BS test, and reduce jump height and average power lossess in the CMJ test produced in response to the BS test.

## Methods

### Experimental design

**Fig. 1:**
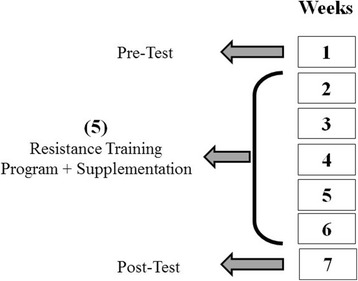
Study design

Participants undertook a 5-week resistance training program during which half the subjects took BA supplements according to whether they were assigned to a placebo group (PLA) or BA group. Before and after the training program, all participants performed a BS incremental load test at the laboratory under the same controlled environmental conditions. During the rest periods of this test, CMJ ability was monitored. The rest period from the pre-training BS test to the start of the training program was 72 h. Similarly, the rest period between the end of the training program and the post-BS test was also 72 h (Fig. 1).

### Subjects

Thirty young, healthy, resistance-trained men were enrolled in the study. All subjects were students of the Physical activity and Sport Sciences degree course at the Universidad Alfonso X El Sabio (Madrid, Spain). Exclusion criteria were: age younger than 18 or older than 25 years; 2) being an elite athlete; 3) having consumed any substance that could affect hormone levels or sport performance in the previous 3 months such as nutrition complements or steroids; 4) having consumed any narcotic and/or psychotropic agents, drugs or stimulants during the test or supplementation period; 5) any cardiovascular, metabolic, neurologic, pulmonary or orthopedic disorder that could limit performance in the different tests; 6) having less than 6 months of experience with BS training; 7) or less than 12 months of experience with resistance training; or 8) having a BS 1RM lower than 90 kg.

Subjects were randomly assigned to the two experimental groups: individuals in one group (*n* = 15) took BA and those in the control group (n = 15) were given placebo supplements (PLA) during the 5 weeks of training. Each day, it was ensured that each subject took the required supplement dose and attended the training sessions. At the end of the study, data were eliminated for subjects not completing all laboratory testing sessions, at least 85% of the training sessions, and/or missing three days or more of supplements. According to these criteria, the final study population was comprised of 26 subjects (age = 21.85 ± 1.6 years; weight = 80.27 ± 6.9 kg; height = 179.62 ± 6.1 cm; body mass index = 24.85 ± 1.8 kg·m^2–1^): 14 in BA and 12 in PLA.

At the study outset, participants were informed of the study protocol, schedule and nature of the exercises and tests to be performed before signing an informed consent form. The study protocol adhered to the tenets of the declaration of Helsinki and was approved by the Ethics Committee of the Universidad Alfonso X El Sabio.

### Supplementation with β-alanine

The authors packaged and prepared the capsules containing the supplement or placebo. The capsules used were no. 1 opaque red (Guinama S.L.U, 0044634, La Pobla de Valbona, Spain) of capacity 0.50 mL/capsule corresponding to 400 mg of BA [34]. For the encapsulation process, we followed the normalized working procedures, *“Procedimientos Normalizados de Trabajo (PNT)”* described for this purpose in the *Formulario Nacional Español*. The filling equipment used was a manual semiautomatic Carsunorm 2000 system (Miranda de Ebro, Spain).

Based on the doses used in other studies [35, 36], subjects assigned to the BA group were administered a daily β-alanine dose of 6.4 g taken as 8 capsules containing 800 mg at least 1.5 h apart and no longer than 3 h apart. The reason for the 8 daily capsules was to avoid the main side effect of paresthesia [4]. Paresthesia is a mild sensation of prickling, numbness or burning in the skin that appears when doses of BA greater than 10 mg/kg are taken [10] and resolves 1 h after intake [10]. Subjects in PLA took the same number of daily capsules containing sucrose. Only one of the authors was responsible for supplying the participants with the corresponding bottles of capsules. All subjects visited the research laboratory weekly to collect their supplement (BA or PLA) for that week. During each of the 5 weeks of the training program, the authors ensured each participant took their supplements and also guided each training session.

### Training program

The 5-week training program was the same for the two groups BA and PLA. Three sessions were conducted per week (15 sessions in total) of around 35–60 min. Each day a register was taken and any participant missing more than 2 sessions (ie, around 15%) was excluded. In each session, after a 15 min warm up, three leg exercises were alternated as a circuit: back squat, barbell step ups and loaded jumping lunges (Table 1). Subjects performed a given number of repetitions of each exercise according to the allocated work time. In the first week, work time was 40 s per exercise and this was reduced by 5 s each week until a work time of 20 s (Table 2). Participants indicated their subjective exertion using the Borg scale of rating of perceived exertion (RPE) (CR-10) when completing each set of exercises and at the end of the session [37].

**Table 1:** Exercises prescribed in the resistance training program

RESISTANCE TRAINING PROGRAM		
3 days a week × 5 weeks		
1	Back squat	
2	Barbell step up	
3	Loaded jumping lunge

**Table 2:** Training prescription week by week

	Week 1	Week 2	Week 3	Week 4	Week 5
Working time (s)	40 s	35 s	30 s	25 s	20 s
Rest between exercises (s)	120 s	105 s	90 s	75 s	60 s
Rest between sets (s)	120 s	105 s	90 s	75 s	60 s
Number of sets	3	4	4	5	5
BS workload	60% 1RM	based on RPE	based on RPE	based on RPE	based on RPE
SU workload	free	based on RPE	based on RPE	based on RPE	based on RPE
LJL workload	free	based on RPE	based on RPE	based on RPE	based on RPE

*BS* back squat, *SU* barbell step up, *LJL* loaded jumping lunge, *1RM* one-repetition maximum, *RPE* rating of perceived exertion, *s* seconds

Load increases were guided by an observer during the program according to the perceived exertion of the previous week. In the first week, the load selected for the BS was 60% of 1RM obtained in the incremental load test before the start of the training program. In contrast, for the barbell step ups and loaded jumping lunges, the load was adjusted by each individual by targeting an RPE of 5–6 to complete 40 s of each exercise, thus maintaining around 50–60% of maximal intensity. Therefore, from the second week onwards: when RPE was 1 point below or above the target, the training load was increased or reduced respectively by 5% (kg) in each exercise; when between 1 and 2.5 points below or above the target, the load was adjusted by 10% (kg); and when 2.5 points above or below the target, the load was adjusted by 15%–20% [38, 39].

To increase the training volume, rest periods between exercises were reduced by 15 s per week from an initial 120 s to 60 s in the fifth week (Table 2). Rest periods between exercise sets were initially 2 min and then reduced by 15 s weekly until 1 min (Table 2). The numbers of exercise sets executed were 3 sets in week 1, 4 sets in weeks 2 and 3, and 5 sets in weeks 4 and 5.

### Pre- and post-training test

#### Warm up

For the pre- and post-training incremental load/CMJ test, subjects first undertook a general warm up followed by a specific warm up. The session commenced with 10 min of light to moderate trotting, 5 min of joint movement and ballistic stretching, and 1 set of 5 BS repetitions with a 20 kg load. During this set, subjects were instructed to increase execution velocity, targeting a velocity close to their maximum velocity in the final repetition. After 30 s of rest, subjects executed 3 consecutive CMJ at submaximal intensity. After 1 min of rest, subjects completed 1 set of 2 BS repetitions with 2 s of rest between repetitions, lifting a 30 kg load at maximum velocity of displacement for optimal muscle activation. After 30 s, subjects executed 2 CMJ at maximal intensity with 10 s of rest between jumps.

#### Back squat incremental load test

Three minutes after the warm-up, subjects started the incremental load BS test with an initial load of 30 kg. This load was increased in each set by 15 kg until average bar displacement velocity measured by a linear position transducer was under 0.7 m/s. Loads were then increased gradually in 1–5 kg steps until the 1RM was accurately determined. When mean velocities were above 0.7 m/s, subjects undertook 2 BS repetitions with a rest period between sets of 3 min. For lower velocities, only one repetition per set was executed with 5 min of rest.

The variables recorded in this session were average velocity (AV), peak velocity (PV), average power (AP), peak power (PP) and the load in kg lifted in the incremental BS 1RM test in which power output is at its maximum (Pmax) as follows [40]:

**Velocity** (m·s^− 1^) = vertical movement of the bar (m) x time (s^− 1^).

**Acceleration** (m·s^− 2^) = vertical bar velocity (m·s^− 1^) x time (s^− 1^).

**Force** (N) = system mass (kg) × vertical acceleration of the bar (m·s^− 2^) + acceleration due to gravity (m·s^− 2^).

**Power** (W) = vertical force (N) × vertical bar velocity (m·s^− 1^). Power was calculated based on barbell velocity and not velocity of the centre of mass of the system [41, 42].

#### Back squat technique

For the BS, the subject stands with feet shoulder-width apart and the barbell placed on top of the shoulder blades with hands clutching the barbell, and then flexes the knees to 120° followed by their extension to the original standing position. Maximal strength, or 1RM, was defined as the maximum load the individual was able to lift with the appropriate exercise action [43].

The test was performed in a multipower, bar-guiding system Smith machine (Matrix, Chácara Alvorada, Brazil) using 20, 10, 5, 2.5 and 1.25 kg discs (Matrix). In this set up, both ends of the barbell are fixed allowing only vertical movement of the bar.

To estimate the execution velocity of each repetition in the incremental load test, we used a linear displacement system (Tendo Weight-lifting Analyzer System, Trencin, Slovak Republic). The cable was attached to one end of the bar to avoid hindering the BS movement. This system allows for measurement of the vertical displacement of the the bar according to the exercise movement and using the system’s software (Tendo Weightlifting analyzer 3.6.15), the device provides bar velocities (average and peak) and powers (average and peak) in the incremental load test [40].

### Jump ability and muscular fatigue

At the start of the rest period for each set of the BS incremental load test, jump capacity was measured in 2 CMJs with 30 s of rest between one jump and the next. The variables jump height, power and take off velocity were measured using a Kistler Quattro Jump contact platform (Kistler Instruments, Winterthur, Switzerland). The CMJ test commences with the subject standing with the legs extended and arms on hips. The subject initiates the jump by bending the knees to ~ 90^0^ (eccentric action) and immediately and synchronously then starts to extend the knees (concentric action) in an explosive movement to attain the maximum height possible. During the jump, the knees should be fully extended and contact with the ground is first made with the toes. Subjects were instructed to keep their hands on the hips during the jump and to avoid any sideways or backward/forward movements.

### Statistical analysis

The effects of BA supplementation on the power output, kilograms lifted and movement velocity in response to the 5 weeks of training were assessed through a general linear model with repeated measures two-way analysis of variance as the Levene’s test revealed the homogeneity of variances of the initial variables and the Shapiro Wilk’s test confirmed their normal distribution. We thus considered an inter-subject factor (PLA, BA) and an intra-subject factor (pre-training, post-training) along with the effects of their interaction.

Although the general linear model with two-way analysis of variance revealed no significant differences between pre-training values for the two study groups, we performed a covariance analysis through a univariate procedure, in which the pre-training values were used as covariates to confirm that the differences observed in the general linear model were not due to differences in pre-training values betwee the PLA and BA groups.

To support the results of the previous analyses, we assessed the effect size of the kilograms lifted and number of sets accomplished. The effect size indicating the difference between means of the dependent variables was calculated using the formula: effect size = post – pre. For this analysis we also used a univariate general linear model.

In addition, the pre- and posttraining power and velocity data recorded at different work intensities in the BS incremental load test were compared through linear or polynomic regression models. We also determined through linear regression, the variables determining jump ability (jump height, average power and take off velocity) for different relative workloads in the BS incremental load test.

In all tests, effect size (*ES*) and statistical power (*SP*) were calculated. The general linear model procedure generates an effect size, known as partial eta squared, categorized as small = 0.01, medium =0.06, large = 0.14 [44]. All data are provided as their means, standard deviation, and 95% confidence intervals (CI) when data are provided as percentages. Percentage improvements were calculated using the equation ([post - pre]/pre X 100). Significance was set at *p* < 0.05. All statistical tests were performed using the software package SSPS version 21.0 (SPSS, Chicago, Ill).

## Results

### Incremental BS test

Significantly greater pre-post training (*time* factor) improvements (*p* < 0.001); were detected in the kilograms lifted at Pmax (*F* = 72.425; *ES* = 0.751; *SP* = 1.000): 15.95% in the PLA group (95% CI, 90.90, 106.52) and 20.17% in the BA group (95% CI, 92.16, 106.62). However, no significant effects (*p* = 0.356) on this variable were observed of the interaction *time* x *group* (*F* = 0.888; *ES* = 0.036; *SP* = 0.148) (Table 3) (Additional file 1). Once analysis of covariance had ruled out an effect of the pre-training variables acting as covariate of the kilograms lifted at Pmax, no significant differences (2.36%, *p* = 0.371) were observed between the two groups (PLA: 95% CI, 99.99, 111.11; BA: 95% CI, 103.74, 114.04) (*F* = 0.832; *ES* = 0.035; *SP* = 0.141) (Additional file 2).

**Table 3:** Effects of the 5-week resistance training program in the PLA and BA groups

Variable	Group	Pre (mean ± SD ± CI)	Post (mean ± SD ± CI)	Post-Pre(u)	Post-Pre (%)	CI (95%)	*p* for Group	*p* for Time	*p* for GroupXTime
Kg at 1RM (kg)	PLA	123.92 ± 18.02 (112.38–135.45)	139.33 ± 15.13 (129.45–149.22)	15.41	12.44%	121.16–142.09	0.484	< 0.001*	0.014*
	BA	124.57 ± 20.42 (113.89–135.25)	148.50 ± 17.73 (139.35–157.65)	23.93	19.21%	126.85–146.22			
AV at 1RM (m·s^−1^)	PLA	0.325 ± 0.073 (0.29–0.36)	0.370 ± 0.125 (0.29–0.45)	0.045	12.16%	0.31–0.39	0.328	0.023*	0.354
	BA	0.324 ± 0.049 (0.29–0.36)	0.426 ± 0.137 (0.35–0.50)	0.102	31.57%	0.34–0.41			
PV at 1RM (m·s^−1^)	PLA	0.844 ± 0.222 (0.71–0.8)	0.951 ± 0.203 (0.86–1.05)	0.107	12.7%	0.80–1.00	0.802	0.044*	0.626
	BA	0.881 ± 0.224 (0.76–1.00)	0.947 ± 0.113 (0.86–1.04)	0.066	7.49%	0.82–1.01			
AP at 1RM (W)	PLA	392.16 ± 87.69 (342.70–441.63)	474.8 ± 104.58 (409.99–539.61)	82.64	21.07%	384.77–482.19	0.185	< 0.001*	0.056
	BA	395.14 ± 78.86 (349.35–440.94)	559.70 ± 112.20 (499.71–619.70)	164.56	41.65%	432.33–522.52			
PP at 1RM (W)	PLA	1159.5 ± 338.91 (939.31–1379.69)	1467.42 ± 334.48 (1297.52–1637.31)	307.92	26.56%	1136.50–1490.42	0.332	< 0.001*	0.774
	BA	1258.79 ± 393.66 (1054.92–1462.64)	1599.64 ± 235.49 (1442.35–1756.94)	300.85	23.89%	1265.38–1593.05			
Kg at Pmax (kg)	PLA	91.42 ± 15.73 (83.02–99.81)	106.00 ± 12.43 (97.71–114.28)	14.58	15.95%	90.90–106.52	0.896	< 0.001*	0.356
	BA	90.29 ± 12.53 (82.51–98.06)	108.50 ± 15.03 (100.83–116.17)	18.21	20.17%	92.16–106.62			
AV at Pmax (m·s^−1^)	PLA	0.735 ± 0.096 (0.69–0.78)	0.698 ± 0.072 (0.66–0.74)	− 0.037	−5.03%	0.68–0.75	0.861	0.373	0.226
	BA	0.710 ± 0.054 (0.67–0.75)	0.716 ± 0.055 (0.68–0.75)	0.006	0.85%	0.68–0.74			
PV at Pmax (m·s^−1^)	PLA	1.289 ± 0.122 (1.22–1.36)	1.246 ± 0.088 (1.19–1.30)	− 0.043	− 3.34%	1.22–1.32	0.497	0.323	0.354
	BA	1.291 ± 0.102 (1.23–1.35)	1.289 ± 0.086 (1.24–1.34)	− 0.002	−0.15%	1.25–1.34			
AP at Pmax (W)	PLA	654.75 ± 113.98 (586.27–723.23)	725.08 ± 106.84 (664.74–785.43)	70.33	10.74%	628.31–751.53	0.904	< 0.001*	0.034*
	BA	631.21 ± 115.73 (567.82–694.61)	758.50 ± 96.33 (702.63–814.37)	127.29	20.17%	637.82–751.90			
PP at Pmax (W)	PLA	1397.25 ± 245.66 (1242.88–1551.62)	1565.75 ± 146.17 (1445.86–1685.63)	168.5	12.06%	1351.76–1611.24	0.494	< 0.001*	0.137
	BA	1408.43 ± 269.93 (1265.51–1551.34)	1673.57 ± 238.06 (1562.58–1784.57)	265.14	18.83%	1420.88–1661.12			
Mean AV (m·s^−1^)	PLA	0.673 ± 0.046 (0.65–0.70)	0.705 ± 0.058 (0.68–0.73)	0.032	4.75%	0.67–0.71	0.988	< 0.005*	0.905
	BA	0.674 ± 0.049 (0.65–0.70)	0.704 ± 0.033 (0.68–0.73)	0.050	5.05%	0.68–0.71			
Mean AP (W)	PLA	506.73 ± 68.56 (458.34–555.21)	589.07 ± 73.10 (543.27–634.87)	82.34	16.25%	502.80–593.04	0.611	< 0.001*	0.383
	BA	514.13 ± 90.68 (469.29–558.97)	612.44 ± 79.92 (570.04–654.84)	98.31	19.12%	521.51–605.06			

*PLA* Placebo, *BA* β-alanine supplementation, *Pmax* Maximun power, *AP* Average power, *PP* Peak power, *AV* average velocity, *PV* Peak velocity, *1RM* one-repetition maximun, *kg* Kilogram, *W* Watts, *m·s*^*−1*^ m·second, * = Significant difference (*p* < 0.05). Data expressed as mean ± standard deviation (SD), ± 95% confidence intervals (CI)

For the variable AP at Pmax, significant effects (*p* < 0.001) were noted of *time* (PLA: 10.74%, 95% CI, 628.31, 751.53; BA: 20.17%, 95% CI, 637.82, 751.90) (*F* = 60.61; *ES* = 0.716; *SP* = 1.000) and of *time* x *group* (*F* = 5.034; *p* = 0.034; *ES* = 0.173; *SP* = 0.577) (Table 3) (Additional file1). When we assessed the covariables, significant differences between groups (4.61%, *p* = 0.037) were confirmed for this variable (PLA: 95% CI, 681.18, 750.46; BA: 95% CI, 734.38, 798.50) (*F* = 4.893; *ES* = 0.175; *SP* = 0.563) (Additional file 2).

In addition, for PP at Pmax, significant differences (*p* < 0.001) were also detected in the factor *time* (*F* = 47.54; *ES* = 0.665; *SP* = 1.000). Improvements were 12.06% (95% CI, 1351.76, 1611.24) and 18.83% (95% CI, 1420.88, 1661.12) for the PLA and BA groups respectively, with no effects of *time* x *group* (*F* = 2.361; *p* = 0.137; *ES* = 0.090; *SP* = 0.314) (Table 3) (Additional file 1).

When we examined factors related to the participants’ 1RM, some significant effects were observed. For the variable kilograms lifted at 1RM differences were significant (*p* < 0.001) for both *time* (PLA: 12.44%, 95% CI, 121.16, 142.09; BA: 19.21%, 95% CI, 126.85, 146.22) (*F* = 151.764; *ES* = 0.863; *SP* = 1.000) and *time* x *group* (*F* = 7.103; *p* = 0.014; *ES* = 0.228; *SP* = 0.725) (Table 3) (Additional file 1). Analysis of covariance confirmed these significant differences between groups (54.42%, *p* = 0.005) eliminating the effect of the covariate pre-training (PLA: 95% CI, 135.40, 143.82; BA: 95% CI, 144.37, 152.16) (*F* = 9.737; *ES* = 0.297; *SP* = 0.848) (Additional file 2).

For AP at 1RM, the *time* factor had a significant effect (*p* < 0.001) (PLA: 21.07%, 95% CI, 384.77, 482.19; BA: 42.65%, 95% CI, 432.33, 522.52) (*F* = 36.862; *ES* = 0.606; *SP* = 1.000) while the impact of *time* x *group* approached significance (*p* = 0.056; *F* = 4.049; *ES* = 0.144; *SP* = 0.489) (Table 3) (Additional file 1). However, by adjusting pre-training levels through analysis of covariance, significant differences (102.42%, *p* = 0.045) were indeed confirmed for AP at 1RM between groups (PLA: 95% CI, 416.26, 535.20; BA: 95% CI, 503.85, 613.97) (*F* = 4.507; *ES* = 0.164; *SP* = 0.529). (Additional file 2).

Significant pre-posttraining differences (*p* < 0.001) were also observed in two last variables related to power, PP at 1RM (PLA: 26.56%, 95% CI, 1136.50, 1490.42; BA: 23.89%, 95% CI, 1265.38, 1593.05) (*F* = 32.797; *ES* = 0.577; *SP* = 1.000) and mean AP (PLA: 16.25%, 95% CI, 502.80, 593.04; BA: 19.12%, 95% CI, 521.51, 605.06) (*F* = 100.680; *ES* = 0.808; *SP* = 1.000). However, no significant effects on these variables of *time* x *group* were noted (*F* = 0.085; *p* = 0.774; *ES* = 0.004; *SP* = 0.059; *F* = 0.791; *p* = 0.383; *ES* = 0.032; *SP* = 0.137, respectively) (Table 3) (Additional file 1). Using as covariates in the univariate ananlysis of variance the pre-training variables, we confirmed the lack of significant differences between BA and PLA for PP at 1RM (− 10.50%, *p* = 0.359, PLA: 95% CI, 1359.53, 1628.13; BA: 95% CI, 1452.75, 1701.25) and mean AP (17.66%, *p* = 0.314, PLA: 95% CI, 566.31, 618.08; BA: 95% CI, 585.80, 633.72) (Additional file 2).

No significant effects were recorded on the variables related to velocity of movement (AV at Pmax, PV at Pmax and peak velocity at 1RM) of either *time* or *group* (Table 3) (Additional file 1).

For mean AV, significant differences (*p* = 0.005) were observed according to *time* (PLA: 95% CI, 0.67, 0.71; BA: 95% CI, 0.67, 0.71) (*F* = 9.529; *ES* = 0.284; *SP* = 0.842), with similar improvements observed in PLA and BA (4.75%, 4.45%, respectively) (Table 3) (Additional file 1).

The following tables (Tables 4 and 5) provide mean pre-post training improvements for BA versus PLA in the number of sets accomplished (*p* = 0.025; 95% CI, 0.82, 2.35, BA: 95% CI, 2.08, 3.49) and number of kilograms lifted (*p* = 0.014; 95% CI, 10.58, 20.25, BA: 95% CI, 19.45, 28.41) in the 1RM test (Additional file 3).

**Table 4:** Mean improvements in the number of sets executed in the pre- versus post-training BS incremental test at 1RM

	Number of repetitions		Sets Post– Sets Pre	CI (95%)	*F/ SP*
	Pre	Post			
Placebo	9.83 ± 1.80	11.41 ± 1.50	1.58 ± 1.44^a^	0.82–2.35	5.709/ 0.630
β-Alanine	10.07 ± 2.26	12.85 ± 1.74	2.79 ± 1.12	2.08–3.49	

^a^significant difference between groups (*p* < 0.05); *SP* statistical power, *CI* confidence interval

**Table 5:** Mean improvements in the number of kilograms lifted in the pre- versus post-training BS incremental test at 1RM

	Kilograms		Kg Post – Kg Pre	CI (95%)	*F/ SP*
	Pre	Post			
Placebo	123.92 ± 18.02	139.33 ± 15.13	15.41 ± 5.82^a^	10.58–20.25	7.103/ 0.725
β-Alanine	124.57 ± 20.42	148.50 ± 17.73	23.92 ± 9.64	19.45–28.41	

^a^significant difference between groups (*p* < 0.05); *SP* statistical power, *CI* confidence interval

**Fig. 2:**
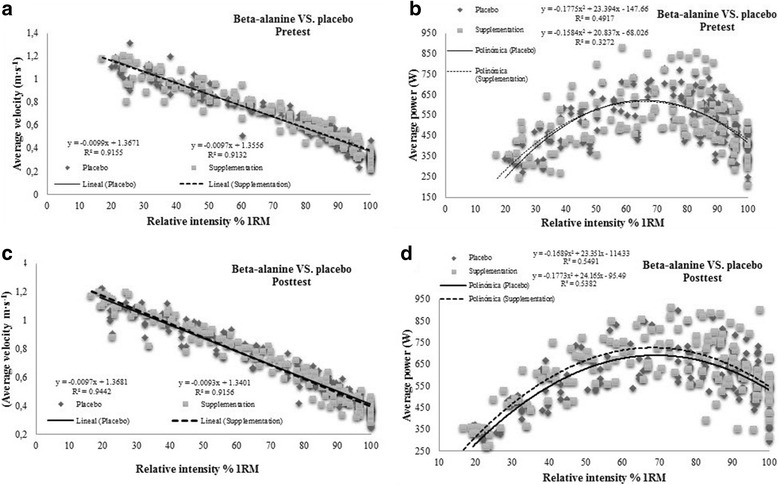
**a** Average velocity β-alanine VS. placebo-Pretest; **b** Average velocity β-alanine VS. placebo-Posttest; **c** Average power β-alanine VS. placebo-Pretest; **d** Average power β-alanine VS. placebo-Posttest

Regression lines for AV recorded in PLA and BA pre- and post-training in the BS incremental load test were similar. This indicates that both 5 weeks of training and supplementation with BA did not modify the relationship between AV and relative work intensity. In contrast, the mean tendency for AP was higher in the BA group than PLA group after training, while means before training failed to vary between the groups, suggesting a beneficial effect of BA supplementation plus training on the BS incremental load test (Fig. 2).

**Fig. 3:**
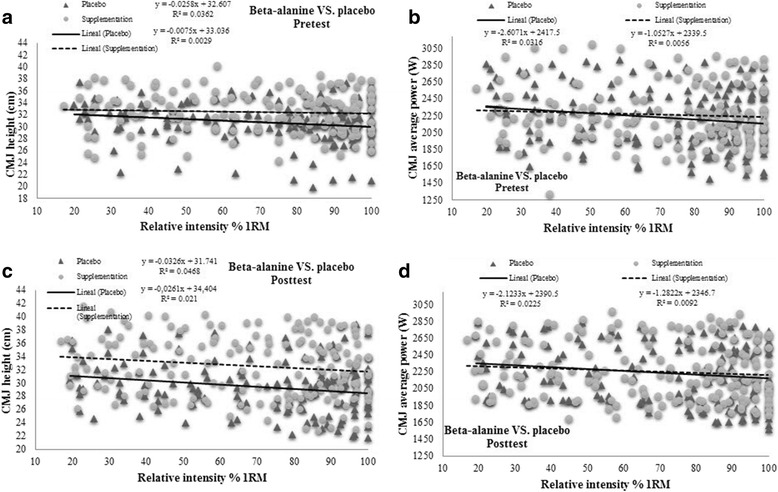
**a** Jump height β-alanine VS. placebo-Pretest; **b** Jump height β-alanine VS. placebo-Posttest; **c** Average power β-alanine VS. placebo-Pretest; **d** Average power β-alanine VS. placebo-Posttest

Regression lines for the variables recorded in the CMJ test, jump height and AP indicated no significant impacts of supplementation during training on these variables (Fig. 3).

## Discussion

In relation to our first hypothesis, the main finding of the present study was a significant improvement produced in AP at 1RM in response to a 5 week training program in the group of subjects who took 6.4 g/day of BA throughout the course of training. This improved average power was attributed to a greater accomplished training load and more kilograms lifted in the BA group, with no differences recorded between groups in movement velocity, thus confirming our second working hypothesis. In response to the third hypothesis, scarce differences between groups were observed in the height and AP values recorded in the CMJ tests despite more kilograms lifted (BA =24 kg, PLA =16 kg) and more sets executed (BA = 2.79 sets, PLA = 1.58 sets) in the incremental BS test after 5 weeks of training in the BA group.

Significant improvements in the kilograms lifted at 1RM in response to the training intervention, were 12.44% (16 kg) for PLA and 19.21% (24 kg) for BA. Similar strength gains (9.3 ± 6.7%) to those observed in our PLA group have been reported in response to a 6-week training intervention in 56 participants in an incremental load test of similar characteristics [45]. In contrast, a greater improvement was observed here in the subjects in our BA supplement group (19.21%) than the gains reported by others [45].

Similar supplementation effects on strength gains have been reported by Hoffman et al. (2006) [19] who observed that both supplementation with creatine and with creatine plus BA was effective at significantly increasing the BS 1RM load (25 kg) over the increase produced with placebo (5 kg) in response to 10 weeks of strength training. A novel finding of our study was that subjects taking BA supplements, besides improving their 1RM, were able to execute significantly more sets in the incremental load test compared to the subjects receiving placebo (2.79 VS. 1.58 sets) (Table 4).

The increase produced in the number of sets completed in the BA group may be related to the pH regulation capacity of BA [46]. This supplement could have had only an indirect ergogenic effect due to the scarce contribution of glycolytic energy metabolism in the incremental exercise used in our study. In other words, the lifts in the test were classed as explosive actions in which energy is mainly provided by the high-energy phosphagen system [18]. Further, the rest periods used in this test were sufficient to replenish the used phosphocreatine reserves, as its resynthesis involves a rapid first stage resulting in the recovery of up to 70% of stores, followed by a second stage extending into minutes 3–5 when reserves have completely recovered [47].

For the half squat, it has been shown that the lactate threshold is reached at work intensities approaching 25% of 1RM [38, 48]. Above this threshold, a glycolytic type metabolism starts to predominate [49]. Thus, the most used energy metabolism during the 5-week training period tested here was glycolytic. Besides their intensity, the duration of the exercise sets (20–40 s) performed here suggests that a lowered pH could limit performance during training sessions. In effect, in a recent study it was noted that BA supplementation improves the number of repetitions performed lifting a load equivalent to 65% of 1RM [20]. These findings indicate that the supplement increases the training session work load [20] and support the results of Hoffman et al. (2006) [19], who observed that BA plus creatine supplementation improved training volume in a strength training program compared to placebo or creatine alone. Thus, the mechanism for this ergogenic effect would involve executing a greater training volume in each pre-post session or improved adaptive responses to the program in the subjects who took BA. This could be observed in the incremental BS test at 1RM, whereby significant improvements were recorded not only in the number of sets undertaken by subjects in the BA group compared to PLA group (2.79 VS. 1.58 sets), but also in the load improvement pre- minus post-training (24 VS. 16 kg) (Table 4).

Muscle power is one of the major determinants of sport performance, and high power levels are required in numerous sport modalities [21, 22]. A common target for athletes is to apply maximum power levels to a given work load. Our results suggest a significantly greater impact on AP at 1RM (*p* = 0.045, 41.70% VS. 21.10%) of BA supplementation than of PLA, possibly explained by the significant improvement recorded in the kilograms lifted at 1RM (*p* = 0.005, 19.21% VS. 12.44%). However, although the gain produced in AP at Pmax was also significantly greater in BA than PLA (*p* = 0.037, 20.17% VS. 10.74%), the improvement in the number of kg lifted at Pmax was not significant (*p* = 0.371, 20.17% VS. 15.95%). These beneficial impacts of supplementation with BA on AP are consistent with observations related to caffeine supplementation [23, 24].

Del Coso et al. (2012) [23] reported that supplementation with a single dose of 3 mg·kg^− 1^ of caffeine was effective at improving average power during an incremental BS test in which loads were increased from 10% to 100% 1RM in steps of 10% 1RM in moderately strength-trained subjects. These findings were confirmed in highly trained subjects in which this same dose of caffeine improved AP levels when lifting loads of 25%, 50% and 75% of 1RM, while higher supplement doses (6 and 9 mg·kg^− 1^) improved AP levels at loads of 25%, 50%, 75% and 90% of 1RM [24]. In both studies, average velocity also increased with each work load [23, 24]. Thus, caffeine supplementation improved AP performance, likely because of the recruitment of more motor units [50].

In contrast with the beneficial effects of caffeine on power output in parallel with barbell displacement velocity, BA supplementation seems to increase power through an increased training volume without affecting the relationship between intensity and velocity. This may be observed in Table 3 and Fig. 2, in which none of the velocity variables differed significantly between the two groups (*p* > 0.05 for AV at Pmax, AV at 1RM, and mean AV). Accordingly, this could indicate different mechanisms underlying the impacts of caffeine and BA on power production. Further work is needed to examine the possibility of a synergistic effect of both supplements in athletes following strength programs targeted at improving power output.

Sodium bicarbonate has also been tested in athletes as the main acid-base regulator and described as superior even to carnosine, which may reduce H^+^ produced through glycolytic pathway activation during high-intensity exercise by up to 62% [51]. The goal of sodium bicarbonate supplementation is to increase plasma bicarbonate levels and thus increase alkaline capacity before an exercise effort with a high anaerobic glycolysis contribution [52]. Given the high glycolytic component of strength training sessions, Carr et al. (2013) [53] administered 300 mg·kg^− 1^of sodium bicarbonate to a group of athletes conducting a typical training session targeting muscle hypertrophy (4 sets of 10–12 maximum repetitions in 3 lower limb exercises). Results indicated that sodium bicarbonate supplementation enabled the execution of a greater training volume. In a second study, it was also observed that sodium bicarbonate supplementation (300 mg·kg^− 1^) was effective at increasing the training volume in strength training sessions as a higher number of repetitions were accomplished in three sets using a load equivalent to 80% of 1RM [54].

These results as well as prior investigations suggest that combining BA and sodium bicarbonate has a synergistic effect that is not observed with each supplement alone. Further, this suggests that sodium bicarbonate might potentiate the effects of BA by increasing training volume and thus promote further adaptations with regards to strength training [17].

In the present study, we also assessed muscular fatigue through performance in a CMJ. No prior work has tested jump ability at the end of each set of an incremental strength test despite being a common laboratory test [27–33]. However, no appreciable pre-posttraining differences were detected between our BA and PLA groups. Hence, jump height and average power values recorded in the CMJ test were similar in both groups despite more kilograms lifted (24 kg VS. 16 kg) and more sets accomplished (2.79 sets VS. 1.58 sets) in the BA supplementation group after 5 weeks of training.

### Limitations

The main limitation of our study was its small sample size (*n* = 26). Four of the subjects enrolled did not fulfil the inclusion requirements as the supplementation and training protocols had to be strictly adhered to. This included a need for 8 doses of 800 mg of supplement (1.5 to 3 h apart) to be taken daily to avoid paresthesia and only two training sessions in the 5 weeks could be missed. The final 26 participants were sufficiently disciplined to complete these requirements of the study design.

### Future lines of research

Based on our findings, future studies should examine the effects of taking both BA and sodium bicarbonate supplements during a strength training program. Further, owing to the effects of BA on work load giving rise to increased power output and to the known benefits of caffeine in improving load displacement velocity in strength training exercises, possible interactions or synergistic effects of caffeine and BA will also need to be explored.

## Conclusions

Five weeks of supplementation with 6.4 g/day of β-alanine compared with placebo during strength training led to increases in: 1) power output for loads equivalent to 1RM; 2) kilograms lifted at 1RM; 3) power output gains at maximum power; 4) the number of sets executed; and 5) the pre-post gain in kilograms lifted at 1RM in an incremental load test.

The ergogenic effects of β-alanine supplementation on power generation were the result of an increased work load. No effects of supplementation were produced on velocity of movement variables or on CMJ test performance (jump height and power).

## Additional files

Additional file 1: General linear model with repeated measures two-way analysis of variance. (PDF 661 kb)

Additional file 2: Covariance analysis throught a univariate procedure. (PDF 261 kb)

Additional file 3: Univariate general linear model. (PDF 169 kb)

## Abbreviations

1RM: One-repetition maximum
AP: Average power
AV: Average velocity
BA: β-alanine
BS: Back squat
CMJ: Countermovement jump
ES: Effect size
PLA: Placebo
Pmax: Maximum power
PP: Peak power
PV: Peak velocity
RPE: Scale of rating of perceived exertion
SP: Statistical power
