# Supplementary material for: Effects of β-alanine supplementation during a 5-week strength training program: a randomized, controlled study
Source: J Int Soc Sports Nutr. 2018 Apr 25;15:19. doi: 10.1186/s12970-018-0224-0 (PMC5918575; doi:10.1186/s12970-018-0224-0)
Supplement: Supplementary file 1 — General linear model with repeated measures two-way analysis of variance. (PDF 661 kb) [file 12970_2018_224_MOESM1_ESM.pdf]

ADDITIONAL FILE 1

Modelo lineal general Medidas repetidas

Kg at Pmax (kg)

Factores intra-sujetos

Medida:MEASURE_1

tiempo	Variable dependiente
1	Kg_Pmax_Pre
2	Kg_Pmax_Pos

Factores inter-sujetos

		Etiqueta del valor	N
Grupo	1,00	Control	12
	2,00	Beta-alanina	14

Estadísticos descriptivos

	Grupo	Media	Desviación típica	N
Kg_Pmax_Pre	Control	91,4167	15,73334	12
	Beta-alanina	90,2857	12,53566	14
	Total	90,8077	13,81888	26
Kg_Pmax_Pos	Control	106,0000	12,43163	12
	Beta-alanina	108,5000	15,03202	14
	Total	107,3462	13,67901	26

Contrastes multivariados[c]

Efecto		Valor	F	Gl de la hipótesis	Gl del error	Sig.	Eta al cuadrado parcial	Parámetro de no centralidad Parámetro	Potencia observada[b]
tiempo	Traza de Pillai	,751	72,425[a]	1,000	24,000	,000	,751	72,425	1,000
	Lambda de Wilks	,249	72,425[a]	1,000	24,000	,000	,751	72,425	1,000
	Traza de Hotelling	3,018	72,425[a]	1,000	24,000	,000	,751	72,425	1,000
	Raíz mayor de Roy	3,018	72,425[a]	1,000	24,000	,000	,751	72,425	1,000
tiempo * Grupo	Traza de Pillai	,036	,888[a]	1,000	24,000	,356	,036	,888	,148
	Lambda de Wilks	,964	,888[a]	1,000	24,000	,356	,036	,888	,148
	Traza de Hotelling	,037	,888[a]	1,000	24,000	,356	,036	,888	,148
	Raíz mayor de Roy	,037	,888[a]	1,000	24,000	,356	,036	,888	,148

a. Estadístico exacto

b. Calculado con alfa = ,05

c. Diseño: Intersección + Grupo

Diseño intra-sujetos: tiempo

Pruebas de efectos intra-sujetos.

Medida:MEASURE_1

Origen		Suma de cuadrados tipo III	gl	Media cuadrática	F	Sig.	Eta al cuadrado parcial	Parámetro de no centralidad Parámetro	Potencia observada[a]
tiempo	Esfericidad asumida	3475,286	1	3475,286	72,425	,000	,751	72,425	1,000
	Greenhouse-Geisser	3475,286	1,000	3475,286	72,425	,000	,751	72,425	1,000
	Huynh-Feldt	3475,286	1,000	3475,286	72,425	,000	,751	72,425	1,000
	Límite-inferior	3475,286	1,000	3475,286	72,425	,000	,751	72,425	1,000
tiempo * Grupo	Esfericidad asumida	42,594	1	42,594	,888	,356	,036	,888	,148
	Greenhouse-Geisser	42,594	1,000	42,594	,888	,356	,036	,888	,148
	Huynh-Feldt	42,594	1,000	42,594	,888	,356	,036	,888	,148
	Límite-inferior	42,594	1,000	42,594	,888	,356	,036	,888	,148
Error(tiempo)	Esfericidad asumida	1151,637	24	47,985					
	Greenhouse-Geisser	1151,637	24,000	47,985					
	Huynh-Feldt	1151,637	24,000	47,985					
	Límite-inferior	1151,637	24,000	47,985					

a. Calculado con alfa = ,05

Contraste de Levene sobre la igualdad de las varianzas error[a]

	F	gl1	gl2	Sig.
Kg_Pmax_Pre	,356	1	24	,557
Kg_Pmax_Pos	,103	1	24	,751

Contrasta la hipótesis nula de que la varianza error de la variable dependiente es igual a lo largo de todos los grupos.

a. Diseño: Intersección + Grupo

Diseño intra-sujetos: tiempo

Pruebas de los efectos inter-sujetos

Medida:MEASURE_1

Variable transformada:Promedio

Origen	Suma de cuadrados tipo III	gl	Media cuadrática	F	Sig.	Eta al cuadrado parcial	Parámetro de no centralidad Parámetro	Potencia observada[a]
Intersección	507154,286	1	507154,286	1475,065	,000	,984	1475,065	1,000
Grupo	6,055	1	6,055	,018	,896	,001	,018	,052
Error	8251,637	24	343,818					

a. Calculado con alfa = ,05

Modelo lineal general Medidas repetidas

AP at Pmax (W)

Factores intra-sujetos

Medida:MEASURE_1

tiempo	Variable dependiente
1	Pm_MaxP_Pre
2	Pm_MaxP_Pos

Factores inter-sujetos

		Etiqueta del valor	N
Grupo	1,00	Control	12
	2,00	Beta-alanina	14

Estadísticos descriptivos

	Grupo	Media	Desviación típica	N
Pm_MaxP_Pre	Control	654,7500	113,98016	12
	Beta-alanina	631,2143	115,73257	14
	Total	642,0769	113,24449	26
Pm_MaxP_Pos	Control	725,0833	106,84352	12
	Beta-alanina	758,5000	96,33336	14
	Total	743,0769	100,68324	26

Contrastes multivariados[c]

Efecto		Valor	F	Gl de la hipótesis	Gl del error	Sig.	Eta al cuadrado parcial	Parámetro de no centralidad Parámetro	Potencia observada[b]
tiempo	Traza de Pillai	,716	60,609[a]	1,000	24,000	,000	,716	60,609	1,000
	Lambda de Wilks	,284	60,609[a]	1,000	24,000	,000	,716	60,609	1,000
	Traza de Hotelling	2,525	60,609[a]	1,000	24,000	,000	,716	60,609	1,000
	Raíz mayor de Roy	2,525	60,609[a]	1,000	24,000	,000	,716	60,609	1,000
tiempo * Grupo	Traza de Pillai	,173	5,034[a]	1,000	24,000	,034	,173	5,034	,577
	Lambda de Wilks	,827	5,034[a]	1,000	24,000	,034	,173	5,034	,577
	Traza de Hotelling	,210	5,034[a]	1,000	24,000	,034	,173	5,034	,577
	Raíz mayor de Roy	,210	5,034[a]	1,000	24,000	,034	,173	5,034	,577

Pruebas de efectos intra-sujetos.

Medida:MEASURE_1

Origen		Suma de cuadrados tipo III	gl	Media cuadrática	F	Sig.	Eta al cuadrado parcial	Parámetro de no centralidad Parámetro	Potencia observada[a]
tiempo	Esfericidad asumida	126172,161	1	126172,161	60,609	,000	,716	60,609	1,000
	Greenhouse-Geisser	126172,161	1,000	126172,161	60,609	,000	,716	60,609	1,000
	Huynh-Feldt	126172,161	1,000	126172,161	60,609	,000	,716	60,609	1,000
	Límite-inferior	126172,161	1,000	126172,161	60,609	,000	,716	60,609	1,000
tiempo * Grupo	Esfericidad asumida	10479,238	1	10479,238	5,034	,034	,173	5,034	,577
	Greenhouse-Geisser	10479,238	1,000	10479,238	5,034	,034	,173	5,034	,577
	Huynh-Feldt	10479,238	1,000	10479,238	5,034	,034	,173	5,034	,577
	Límite-inferior	10479,238	1,000	10479,238	5,034	,034	,173	5,034	,577
Error(tiempo)	Esfericidad asumida	49961,762	24	2081,740					
	Greenhouse-Geisser	49961,762	24,000	2081,740					
	Huynh-Feldt	49961,762	24,000	2081,740					
	Límite-inferior	49961,762	24,000	2081,740					

a. Calculado con alfa = ,05

Contraste de Levene sobre la igualdad de las varianzas error[a]

	F	gl1	gl2	Sig.
Pm_MaxP_Pre	,008	1	24	,930
Pm_MaxP_Pos	,116	1	24	,737

Pruebas de los efectos inter-sujetos

Medida:MEASURE_1

Variable transformada:Promedio

Origen	Suma de cuadrados tipo III	gl	Media cuadrática	F	Sig.	Eta al cuadrado parcial	Parámetro de no centralidad Parámetro	Potencia observada[a]
Intersección	2,478E7	1	2,478E7	1158,727	,000	,980	1158,727	1,000
Grupo	315,430	1	315,430	,015	,904	,001	,015	,052
Error	513279,262	24	21386,636					

a. Calculado con alfa = ,05

Modelo lineal general Medidas repetidas

PP at Pmax (W)

Factores intra-sujetos

Medida:MEASURE_1

tiempo	Variable dependiente
1	Pp_MaxP_Pre
2	Pp_MaxP_Pos

Factores inter-sujetos

		Etiqueta del valor	N
Grupo	1,00	Control	12
	2,00	Beta-alanina	14

Estadísticos descriptivos

	Grupo	Media	Desviación típica	N
Pp_MaxP_Pre	Control	1397,2500	245,66242	12
	Beta-alanina	1408,4286	269,93353	14
	Total	1403,2692	253,92055	26
Pp_MaxP_Pos	Control	1565,7500	146,16872	12
	Beta-alanina	1673,5714	238,06131	14
	Total	1623,8077	204,63509	26

Contrastes multivariados[c]

Efecto		Valor	F	Gl de la hipótesis	Gl del error	Sig.	Eta al cuadrado parcial	Parámetro de no centralidad Parámetro	Potencia observada[b]
tiempo	Traza de Pillai	,665	47,545[a]	1,000	24,000	,000	,665	47,545	1,000
	Lambda de Wilks	,335	47,545[a]	1,000	24,000	,000	,665	47,545	1,000
	Traza de Hotelling	1,981	47,545[a]	1,000	24,000	,000	,665	47,545	1,000
	Raíz mayor de Roy	1,981	47,545[a]	1,000	24,000	,000	,665	47,545	1,000
tiempo * Grupo	Traza de Pillai	,090	2,361[a]	1,000	24,000	,137	,090	2,361	,314
	Lambda de Wilks	,910	2,361[a]	1,000	24,000	,137	,090	2,361	,314
	Traza de Hotelling	,098	2,361[a]	1,000	24,000	,137	,090	2,361	,314
	Raíz mayor de Roy	,098	2,361[a]	1,000	24,000	,137	,090	2,361	,314

a. Estadístico exacto

b. Calculado con alfa = ,05

c. Diseño: Intersección + Grupo

Diseño intra-sujetos: tiempo

Contraste de Levene sobre la igualdad de las varianzas error[a]

	F	gl1	gl2	Sig.
Pp_MaxP_Pre	,003	1	24	,956
Pp_MaxP_Pos	1,305	1	24	,265

Pruebas de los efectos inter-sujetos

Medida:MEASURE_1

Variable transformada:Promedio

Origen	Suma de cuadrados tipo III	gl	Media cuadrática	F	Sig.	Eta al cuadrado parcial	Parámetro de no centralidad Parámetro	Potencia observada[a]
Intersección	1,181E8	1	1,181E8	1244,810	,000	,981	1244,810	1,000
Grupo	45750,923	1	45750,923	,482	,494	,020	,482	,102
Error	2276181,000	24	94840,875					

a. Calculado con alfa = ,05

Modelo lineal general Medidas repetidas

Kg at 1RM (kg)

Factores intra-sujetos

Medida:MEASURE_1

tiempo	Variable dependiente
1	RM_kg_Pre
2	RM_kg_Pos

Factores inter-sujetos

		Etiqueta del valor	N
Grupo	1,00	Control	12
	2,00	Beta-alanina	14

Estadísticos descriptivos

	Grupo	Media	Desviación típica	N
RM_kg_Pre	Control	123,9167	18,02250	12
	Beta-alanina	124,5714	20,42139	14
	Total	124,2692	18,97063	26
RM_kg_Pos	Control	139,3333	15,12574	12
	Beta-alanina	148,5000	17,73198	14
	Total	144,2692	16,90812	26

Contrastes multivariados[c]

Efecto		Valor	F	Gl de la hipótesis	Gl del error	Sig.	Eta al cuadrado parcial	Parámetro de no centralidad Parámetro	Potencia observada[b]
tiempo	Traza de Pillai	,863	151,764[a]	1,000	24,000	,000	,863	151,764	1,000
	Lambda de Wilks	,137	151,764[a]	1,000	24,000	,000	,863	151,764	1,000
	Traza de Hotelling	6,323	151,764[a]	1,000	24,000	,000	,863	151,764	1,000
	Raíz mayor de Roy	6,323	151,764[a]	1,000	24,000	,000	,863	151,764	1,000
tiempo * Grupo	Traza de Pillai	,228	7,103[a]	1,000	24,000	,014	,228	7,103	,725
	Lambda de Wilks	,772	7,103[a]	1,000	24,000	,014	,228	7,103	,725
	Traza de Hotelling	,296	7,103[a]	1,000	24,000	,014	,228	7,103	,725
	Raíz mayor de Roy	,296	7,103[a]	1,000	24,000	,014	,228	7,103	,725

a. Estadístico exacto
b. Calculado con alfa = ,05
c. Diseño: Intersección + Grupo
Diseño intra-sujetos: tiempo

Contraste de Levene sobre la igualdad de las varianzas error[a]

	F	gl1	gl2	Sig.
RM_kg_Pre	,041	1	24	,841
RM_kg_Pos	,299	1	24	,589

Pruebas de los efectos inter-sujetos

Medida:MEASURE_1

Variable transformada:Promedio

Origen	Suma de cuadrados tipo III	gl	Media cuadrática	F	Sig.	Eta al cuadrado parcial	Parámetro de no centralidad Parámetro	Potencia observada[a]
Intersección	929300,641	1	929300,641	1506,202	,000	,984	1506,202	1,000
Grupo	311,641	1	311,641	,505	,484	,021	,505	,105
Error	14807,589	24	616,983					

Contraste de Levene sobre la igualdad de las varianzas error[a]

	F	gl1	gl2	Sig.
RM_kg_Pre	,041	1	24	,841
RM_kg_Pos	,299	1	24	,589

Pruebas de los efectos inter-sujetos

Medida:MEASURE_1

Variable transformada:Promedio

Origen	Suma de cuadrados tipo III	gl	Media cuadrática	F	Sig.	Eta al cuadrado parcial	Parámetro de no centralidad Parámetro	Potencia observada[a]
Intersección	929300,641	1	929300,641	1506,202	,000	,984	1506,202	1,000
Grupo	311,641	1	311,641	,505	,484	,021	,505	,105
Error	14807,589	24	616,983					

a. Calculado con alfa = ,05

Modelo lineal general Medidas repetidas

AP at 1RM (W)

Factores intra-sujetos

Medida:MEASURE_1

tiempo	Variable dependiente
1	RM_Pm_Pre
2	RM_Pm_Pos

Factores inter-sujetos

		Etiqueta del valor	N
Grupo	1,00	Control	12
	2,00	Beta-alanina	14

Estadísticos descriptivos

	Grupo	Media	Desviación típica	N
RM_Pm_Pre	Control	392,1667	87,68522	12
	Beta-alanina	395,1429	78,85806	14
	Total	393,7692	81,35714	26
RM_Pm_Pos	Control	474,8000	104,57919	12
	Beta-alanina	559,7050	112,19876	14
	Total	520,5181	114,98461	26

Contrastes multivariados[c]

Efecto		Valor	F	Gl de la hipótesis	Gl del error	Sig.	Eta al cuadrado parcial	Parámetro de no centralidad Parámetro	Potencia observada[b]
tiempo	Traza de Pillai	,606	36,862[a]	1,000	24,000	,000	,606	36,862	1,000
	Lambda de Wilks	,394	36,862[a]	1,000	24,000	,000	,606	36,862	1,000
	Traza de Hotelling	1,536	36,862[a]	1,000	24,000	,000	,606	36,862	1,000
	Raíz mayor de Roy	1,536	36,862[a]	1,000	24,000	,000	,606	36,862	1,000
tiempo * Grupo	Traza de Pillai	,144	4,049[a]	1,000	24,000	,056	,144	4,049	,489
	Lambda de Wilks	,856	4,049[a]	1,000	24,000	,056	,144	4,049	,489
	Traza de Hotelling	,169	4,049[a]	1,000	24,000	,056	,144	4,049	,489
	Raíz mayor de Roy	,169	4,049[a]	1,000	24,000	,056	,144	4,049	,489

Contraste de Levene sobre la igualdad de las varianzas error[a]

	F	gl1	gl2	Sig.
RM_Pm_Pre	,392	1	24	,537
RM_Pm_Pos	,004	1	24	,953

Contrasta la hipótesis nula de que la varianza error de la variable dependiente es igual a lo largo de todos los grupos.

a. Diseño: Intersección + Grupo

Diseño intra-sujetos: tiempo

Pruebas de los efectos inter-sujetos

Medida:MEASURE_1

Variable transformada:Promedio

Origen	Suma de cuadrados tipo III	gl	Media cuadrática	F	Sig.	Eta al cuadrado parcial	Parámetro de no centralidad Parámetro	Potencia observada[a]
Intersección	1,072E7	1	1,072E7	802,118	,000	,971	802,118	1,000
Grupo	24951,566	1	24951,566	1,866	,185	,072	1,866	,259
Error	320839,155	24	13368,298					

a. Calculado con alfa = ,05

Modelo lineal general Medidas repetidas

PP at 1RM (W)

Factores intra-sujetos

Medida:MEASURE_1

tiempo	Variable dependiente
1	RM_Pp_Pre
2	RM_Pp_Pos

Factores inter-sujetos

		Etiqueta del valor	N
Grupo	1,00	Control	12
	2,00	Beta-alanina	14

Estadísticos descriptivos

	Grupo	Media	Desviación típica	N
RM_Pp_Pre	Control	1159,5000	338,91069	12
	Beta-alanina	1258,7857	393,66369	14
	Total	1212,9615	365,61099	26
RM_Pp_Pos	Control	1467,4167	334,48263	12
	Beta-alanina	1599,6429	235,48856	14
	Total	1538,6154	287,37085	26

Contrastes multivariados[c]

Efecto		Valor	F	Gl de la hipótesis	Gl del error	Sig.	Eta al cuadrado parcial	Parámetro de no centralidad Parámetro	Potencia observada[b]
tiempo	Traza de Pillai	,577	32,797[a]	1,000	24,000	,000	,577	32,797	1,000
	Lambda de Wilks	,423	32,797[a]	1,000	24,000	,000	,577	32,797	1,000
	Traza de Hotelling	1,367	32,797[a]	1,000	24,000	,000	,577	32,797	1,000
	Raíz mayor de Roy	1,367	32,797[a]	1,000	24,000	,000	,577	32,797	1,000
tiempo * Grupo	Traza de Pillai	,004	,085[a]	1,000	24,000	,774	,004	,085	,059
	Lambda de Wilks	,996	,085[a]	1,000	24,000	,774	,004	,085	,059
	Traza de Hotelling	,004	,085[a]	1,000	24,000	,774	,004	,085	,059
	Raíz mayor de Roy	,004	,085[a]	1,000	24,000	,774	,004	,085	,059

Contraste de Levene sobre la igualdad de las varianzas error[a]

	F	gl1	gl2	Sig.
RM_Pp_Pre	,099	1	24	,756
RM_Pp_Pos	1,172	1	24	,290

Contrasta la hipótesis nula de que la varianza error de la variable dependiente es igual a lo largo de todos los grupos.

a. Diseño: Intersección + Grupo

Diseño intra-sujetos: tiempo

Pruebas de los efectos inter-sujetos

Medida:MEASURE_1

Variable transformada:Promedio

Origen	Suma de cuadrados tipo III	gl	Media cuadrática	F	Sig.	Eta al cuadrado parcial	Parámetro de no centralidad Parámetro	Potencia observada[a]
Intersección	9,721E7	1	9,721E7	550,957	,000	,958	550,957	1,000
Grupo	173162,000	1	173162,000	,981	,332	,039	,981	,158
Error	4234553,173	24	176439,716					

a. Calculado con alfa = ,05

Modelo lineal general Medidas repetidas

Mean AP (W)

Factores intra-sujetos

Medida:MEASURE_1

tiempo	Variable dependiente
1	Media_Pm_Pre
2	Media_Pm_Pos

Factores inter-sujetos

		Etiqueta del valor	N
Grupo	1,00	Control	12
	2,00	Beta-alanina	14

Estadísticos descriptivos

	Grupo	Media	Desviación típica	N
Media_Pm_Pre	Control	506,7733	68,55924	12
	Beta-alanina	514,1329	90,68144	14
	Total	510,7362	79,73823	26
Media_Pm_Pos	Control	589,0700	73,09566	12
	Beta-alanina	612,4407	79,91885	14
	Total	601,6542	76,24520	26

Contrastes multivariados[c]

Efecto		Valor	F	Gl de la hipótesis	Gl del error	Sig.	Eta al cuadrado parcial	Parámetro de no centralidad Parámetro	Potencia observada[b]
tiempo	Traza de Pillai	,808	100,680[a]	1,000	24,000	,000	,808	100,680	1,000
	Lambda de Wilks	,192	100,680[a]	1,000	24,000	,000	,808	100,680	1,000
	Traza de Hotelling	4,195	100,680[a]	1,000	24,000	,000	,808	100,680	1,000
	Raíz mayor de Roy	4,195	100,680[a]	1,000	24,000	,000	,808	100,680	1,000
tiempo * Grupo	Traza de Pillai	,032	,791[a]	1,000	24,000	,383	,032	,791	,137
	Lambda de Wilks	,968	,791[a]	1,000	24,000	,383	,032	,791	,137
	Traza de Hotelling	,033	,791[a]	1,000	24,000	,383	,032	,791	,137
	Raíz mayor de Roy	,033	,791[a]	1,000	24,000	,383	,032	,791	,137

a. Estadístico exacto

b. Calculado con alfa = ,05

c. Diseño: Intersección + Grupo

Diseño intra-sujetos: tiempo

Modelo lineal general Medidas repetidas

AV at Pmax ($m \cdot s^{-1}$)

Factores intra-sujetos

Medida:MEASURE_1

tiempo	Variable dependiente
1	Vm_MaxP_Pre
2	Vm_MaxP_Pos

Factores inter-sujetos

		Etiqueta del valor	N
Grupo	1,00	Control	12
	2,00	Beta-alanina	14

Estadísticos descriptivos

	Grupo	Media	Desviación típica	N
Vm_MaxP_Pre	Control	,7350	,09577	12
	Beta-alanina	,7100	,05378	14
	Total	,7215	,07551	26
Vm_MaxP_Pos	Control	,6983	,07171	12
	Beta-alanina	,7157	,05515	14
	Total	,7077	,06263	26

Contrastes multivariados[c]

Efecto		Valor	F	Gl de la hipótesis	Gl del error	Sig.	Eta al cuadrado parcial	Parámetro de no centralidad Parámetro	Potencia observada[b]
tiempo	Traza de Pillai	,033	,824[a]	1,000	24,000	,373	,033	,824	,141
	Lambda de Wilks	,967	,824[a]	1,000	24,000	,373	,033	,824	,141
	Traza de Hotelling	,034	,824[a]	1,000	24,000	,373	,033	,824	,141
	Raíz mayor de Roy	,034	,824[a]	1,000	24,000	,373	,033	,824	,141
tiempo * Grupo	Traza de Pillai	,060	1,544[a]	1,000	24,000	,226	,060	1,544	,222
	Lambda de Wilks	,940	1,544[a]	1,000	24,000	,226	,060	1,544	,222
	Traza de Hotelling	,064	1,544[a]	1,000	24,000	,226	,060	1,544	,222
	Raíz mayor de Roy	,064	1,544[a]	1,000	24,000	,226	,060	1,544	,222

a. Estadístico exacto

b. Calculado con alfa = ,05

c. Diseño: Intersección + Grupo

Diseño intra-sujetos: tiempo

Contraste de Levene sobre la igualdad de las varianzas error[a]

	F	gl1	gl2	Sig.
Vm_MaxP_Pre	3,538	1	24	,072
Vm_MaxP_Pos	,586	1	24	,452

Pruebas de los efectos inter-sujetos

Medida:MEASURE_1

Variable transformada:Promedio

Origen	Suma de cuadrados tipo III	gl	Media cuadrática	F	Sig.	Eta al cuadrado parcial	Parámetro de no centralidad Parámetro	Potencia observada[a]
Intersección	26,409	1	26,409	4389,130	,000	,995	4389,130	1,000
Grupo	,000	1	,000	,031	,861	,001	,031	,053
Error	,144	24	,006					

a. Calculado con alfa = ,05

Modelo lineal general Medidas repetidas

PV at Pmax ($m \cdot s^{-1}$)

Factores intra-sujetos

Medida:MEASURE_1

tiempo	Variable dependiente
1	Vp_MaxP_Pre
2	Vp_MaxP_Pos

Factores inter-sujetos

		Etiqueta del valor	N
Grupo	1,00	Control	12
	2,00	Beta-alanina	14

Estadísticos descriptivos

	Grupo	Media	Desviación típica	N
Vp_MaxP_Pre	Control	1,2892	,12243	12
	Beta-alanina	1,2907	,10209	14
	Total	1,2900	,10962	26
Vp_MaxP_Pos	Control	1,2458	,08806	12
	Beta-alanina	1,2893	,08606	14
	Total	1,2692	,08804	26

Contrastes multivariados[c]

Efecto		Valor	F	Gl de la hipótesis	Gl del error	Sig.	Eta al cuadrado parcial	Parámetro de no centralidad Parámetro	Potencia observada[b]
tiempo	Traza de Pillai	,041	1,019[a]	1,000	24,000	,323	,041	1,019	,163
	Lambda de Wilks	,959	1,019[a]	1,000	24,000	,323	,041	1,019	,163
	Traza de Hotelling	,042	1,019[a]	1,000	24,000	,323	,041	1,019	,163
	Raíz mayor de Roy	,042	1,019[a]	1,000	24,000	,323	,041	1,019	,163
tiempo * Grupo	Traza de Pillai	,036	,893[a]	1,000	24,000	,354	,036	,893	,148
	Lambda de Wilks	,964	,893[a]	1,000	24,000	,354	,036	,893	,148
	Traza de Hotelling	,037	,893[a]	1,000	24,000	,354	,036	,893	,148
	Raíz mayor de Roy	,037	,893[a]	1,000	24,000	,354	,036	,893	,148

a. Estadístico exacto

b. Calculado con alfa = ,05

c. Diseño: Intersección + Grupo

Contraste de Levene sobre la igualdad de las varianzas error[a]

	F	gl1	gl2	Sig.
Vp_MaxP_Pre	,297	1	24	,591
Vp_MaxP_Pos	,001	1	24	,971

Contrasta la hipótesis nula de que la varianza error de la variable dependiente es igual a lo largo de todos los grupos.

a. Diseño: Intersección + Grupo

Pruebas de los efectos inter-sujetos

Medida:MEASURE_1

Variable transformada:Promedio

Origen	Suma de cuadrados tipo III	gl	Media cuadrática	F	Sig.	Eta al cuadrado parcial	Parámetro de no centralidad Parámetro	Potencia observada[a]
Intersección	84,527	1	84,527	6155,837	,000	,996	6155,837	1,000
Grupo	,007	1	,007	,476	,497	,019	,476	,102
Error	,330	24	,014					

Contrastes multivariados[c]

Efecto		Valor	F	Gl de la hipótesis	Gl del error	Sig.	Eta al cuadrado parcial	Parámetro de no centralidad Parámetro	Potencia observada[b]
tiempo	Traza de Pillai	,041	1,019[a]	1,000	24,000	,323	,041	1,019	,163
	Lambda de Wilks	,959	1,019[a]	1,000	24,000	,323	,041	1,019	,163
	Traza de Hotelling	,042	1,019[a]	1,000	24,000	,323	,041	1,019	,163
	Raíz mayor de Roy	,042	1,019[a]	1,000	24,000	,323	,041	1,019	,163
tiempo * Grupo	Traza de Pillai	,036	,893[a]	1,000	24,000	,354	,036	,893	,148
	Lambda de Wilks	,964	,893[a]	1,000	24,000	,354	,036	,893	,148
	Traza de Hotelling	,037	,893[a]	1,000	24,000	,354	,036	,893	,148
	Raíz mayor de Roy	,037	,893[a]	1,000	24,000	,354	,036	,893	,148

a. Estadístico exacto

b. Calculado con alfa = ,05

a. Calculado con alfa = ,05

Modelo lineal general Medidas repetidas

PV at 1RM ($m \cdot s^{-1}$)

Factores intra-sujetos

Medida:MEASURE_1

tiempo	Variable dependiente
1	RM_Vp_Pre
2	RM_Vp_Pos

Factores inter-sujetos

		Etiqueta del valor	N
Grupo	1,00	Control	12
	2,00	Beta-alanina	14

Estadísticos descriptivos

	Grupo	Media	Desviación típica	N
RM_Vp_Pre	Control	,8442	,22224	12
	Beta-alanina	,8807	,22434	14
	Total	,8638	,21966	26
RM_Vp_Pos	Control	,9508	,20318	12
	Beta-alanina	,9471	,11262	14
	Total	,9488	,15736	26

Contrastes multivariados[c]

Efecto		Valor	F	Gl de la hipótesis	Gl del error	Sig.	Eta al cuadrado parcial	Parámetro de no centralidad Parámetro	Potencia observada[b]
tiempo	Traza de Pillai	,158	4,498[a]	1,000	24,000	,044	,158	4,498	,530
	Lambda de Wilks	,842	4,498[a]	1,000	24,000	,044	,158	4,498	,530
	Traza de Hotelling	,187	4,498[a]	1,000	24,000	,044	,158	4,498	,530
	Raíz mayor de Roy	,187	4,498[a]	1,000	24,000	,044	,158	4,498	,530
tiempo * Grupo	Traza de Pillai	,010	,243[a]	1,000	24,000	,626	,010	,243	,076
	Lambda de Wilks	,990	,243[a]	1,000	24,000	,626	,010	,243	,076
	Traza de Hotelling	,010	,243[a]	1,000	24,000	,626	,010	,243	,076
	Raíz mayor de Roy	,010	,243[a]	1,000	24,000	,626	,010	,243	,076

a. Estadístico exacto

b. Calculado con alfa = ,05

c. Diseño: Intersección + Grupo

Contraste de Levene sobre la igualdad de las varianzas error[a]

	F	gl1	gl2	Sig.
RM_Vp_Pre	,053	1	24	,821
RM_Vp_Pos	2,813	1	24	,107

Contrasta la hipótesis nula de que la varianza error de la variable dependiente es igual a lo largo de todos los grupos.

Pruebas de los efectos inter-sujetos

Medida:MEASURE_1

Variable transformada:Promedio

Origen	Suma de cuadrados tipo III	gl	Media cuadrática	F	Sig.	Eta al cuadrado parcial	Parámetro de no centralidad Parámetro	Potencia observada[a]
Intersección	42,404	1	42,404	782,805	,000	,970	782,805	1,000
Grupo	,003	1	,003	,064	,802	,003	,064	,057
Error	1,300	24	,054					

a. Calculado con alfa = ,05

Modelo lineal general Medidas repetidas

Mean AV (m·s^{-1})

Factores intra-sujetos

Medida:MEASURE_1

tiempo	Variable dependiente
1	Media_Vm_Pre
2	Media_Vm_Pos

Factores inter-sujetos

		Etiqueta del valor	N
Grupo	1,00	Control	12
	2,00	Beta-alanina	14

Estadísticos descriptivos

	Grupo	Media	Desviación típica	N
Media_Vm_Pre	Control	,6733	,04579	12
	Beta-alanina	,6743	,04863	14
	Total	,6738	,04640	26
Media_Vm_Pos	Control	,7050	,05823	12
	Beta-alanina	,7036	,03296	14
	Total	,7042	,04536	26

Contrastes multivariados[c]

Efecto		Valor	F	Gl de la hipótesis	Gl del error	Sig.	Eta al cuadrado parcial	Parámetro de no centralidad Parámetro	Potencia observada[b]
tiempo	Traza de Pillai	,284	9,529[a]	1,000	24,000	,005	,284	9,529	,842
	Lambda de Wilks	,716	9,529[a]	1,000	24,000	,005	,284	9,529	,842
	Traza de Hotelling	,397	9,529[a]	1,000	24,000	,005	,284	9,529	,842
	Raíz mayor de Roy	,397	9,529[a]	1,000	24,000	,005	,284	9,529	,842
tiempo * Grupo	Traza de Pillai	,001	,015[a]	1,000	24,000	,905	,001	,015	,052
	Lambda de Wilks	,999	,015[a]	1,000	24,000	,905	,001	,015	,052
	Traza de Hotelling	,001	,015[a]	1,000	24,000	,905	,001	,015	,052
	Raíz mayor de Roy	,001	,015[a]	1,000	24,000	,905	,001	,015	,052

a. Estadístico exacto

b. Calculado con alfa = ,05

c. Diseño: Intersección + Grupo

Diseño intra-sujetos: tiempo

Pruebas de los efectos inter-sujetos

Medida:MEASURE_1

Variable transformada:Promedio

Origen	Suma de cuadrados tipo III	gl	Media cuadrática	F	Sig.	Eta al cuadrado parcial	Parámetro de no centralidad Parámetro	Potencia observada[a]
Intersección	24,543	1	24,543	7853,576	,000	,997	7853,576	1,000
Grupo	7,326E-7	1	7,326E-7	,000	,988	,000	,000	,050
Error	,075	24	,003					

a. Calculado con alfa = ,05

Modelo lineal general Medidas repetidas

AV at 1RM ($m \cdot s^{-1}$)

Factores intra-sujetos

Medida:MEASURE_1

Tiempo	Variable dependiente
1	RM_Vm_Pre
2	RM_Vm_Pos

Factores inter-sujetos

		Etiqueta del valor	N
Grupo	1,00	Control	12
	2,00	Beta-alanina	14

Estadísticos descriptivos

	Grupo	Media	Desviación típica	N
RM_Vm_Pre	Control	,3250	,07317	12
	Beta-alanina	,3236	,04986	14
	Total	,3242	,06041	26
RM_Vm_Pos	Control	,3700	,12548	12
	Beta-alanina	,4257	,13799	14
	Total	,4000	,13279	26

Contrastes multivariados[c]

Efecto		Valor	F	Gl de la hipótesis	Gl del error	Sig.	Eta al cuadrado parcial	Parámetro de no centralidad Parámetro	Potencia observada[b]
Tiempo	Traza de Pillai	,198	5,929[a]	1,000	24,000	,023	,198	5,929	,647
	Lambda de Wilks	,802	5,929[a]	1,000	24,000	,023	,198	5,929	,647
	Traza de Hotelling	,247	5,929[a]	1,000	24,000	,023	,198	5,929	,647
	Raíz mayor de Roy	,247	5,929[a]	1,000	24,000	,023	,198	5,929	,647
Tiempo * Grupo	Traza de Pillai	,036	,894[a]	1,000	24,000	,354	,036	,894	,149
	Lambda de Wilks	,964	,894[a]	1,000	24,000	,354	,036	,894	,149
	Traza de Hotelling	,037	,894[a]	1,000	24,000	,354	,036	,894	,149
	Raíz mayor de Roy	,037	,894[a]	1,000	24,000	,354	,036	,894	,149

a. Estadístico exacto

b. Calculado con alfa = ,05

c. Diseño: Intersección + Grupo
 Diseño intra-sujetos: Tiempo

Contraste de Levene sobre la igualdad de las varianzas error[a]

	F	gl1	gl2	Sig.
RM_Vm_Pre	1,437	1	24	,242
RM_Vm_Pos	,359	1	24	,555

Contrasta la hipótesis nula de que la varianza error de la variable dependiente es igual a lo largo de todos los grupos.

a. Diseño: Intersección + Grupo
 Diseño intra-sujetos: Tiempo

Pruebas de los efectos inter-sujetos

Medida:MEASURE_1

Variable transformada:Promedio

Origen	Suma de cuadrados tipo III	gl	Media cuadrática	F	Sig.	Eta al cuadrado parcial	Parámetro de no centralidad Parámetro	Potencia observada[a]
Intersección	6,739	1	6,739	706,926	,000	,967	706,926	1,000
Grupo	,010	1	,010	,999	,328	,040	,999	,160
Error	,229	24	,010					

a. Calculado con alfa = ,05
