# Supplementary material for: Effects of β-alanine supplementation during a 5-week strength training program: a randomized, controlled study
Source: J Int Soc Sports Nutr. 2018 Apr 25;15:19. doi: 10.1186/s12970-018-0224-0 (PMC5918575; doi:10.1186/s12970-018-0224-0)
Supplement: Supplementary file 2 — Covariance analysis throught a univariate procedure. (PDF 261 kb) [file 12970_2018_224_MOESM2_ESM.pdf]

ADDITIONAL FILE 2

Análisis de varianza univariante con convariable *Kg_Pmax_Pos*

Factores inter-sujetos

		Etiqueta del valor	N
Grupo	1,00	Control	12
	2,00	Beta-alanina	14

Estadísticos descriptivos

Variable dependiente: ***Kg_Pmax_Pos***

Grupo	Media	Desviación típica	N
Control	106,0000	12,43163	12
Beta-alanina	108,5000	15,03202	14
Total	107,3462	13,67901	26

Contraste de Levene sobre la igualdad de las varianzas error[a]

Variable dependiente:Kg_Pmax_Pos

F	gl1	gl2	Sig.
,930	1	24	,344

Contrasta la hipótesis nula de que la varianza error de la variable dependiente es igual a lo largo de todos los grupos.
a. Diseño: Intersección + Kg_Pmax_Pre + Grupo

Pruebas de los efectos inter-sujetos

Variable dependiente:Kg_Pmax_Pos

Origen	Suma de cuadrados tipo III	gl	Media cuadrática	F	Sig.	Eta al cuadrado parcial	Parámetro de no centralidad Parámetro	Potencia observada[b]
Modelo corregido	2684,761[a]	2	1342,381	15,491	,000	,574	30,981	,998
Intersección	884,562	1	884,562	10,208	,004	,307	10,208	,864
Kg_Pmax_Pre	2644,376	1	2644,376	30,515	,000	,570	30,515	1,000
Grupo	72,063	1	72,063	,832	,371	,035	,832	,141
Error	1993,124	23	86,658					
Total	304281,000	26						
Total corregida	4677,885	25						

a. R cuadrado = ,574 (R cuadrado corregida = ,537)
b. Calculado con alfa = ,05

Medias marginales estimadas

Grupo

Variable dependiente:Kg_Pmax_Pos

Grupo	Media	Error típ.	Intervalo de confianza 95%	
			Límite inferior	Límite superior
Control	105,546[a]	2,689	99,985	111,108
Beta-alanina	108,889[a]	2,489	103,740	114,038

a. Las covariables que aparecen en el modelo se evalúan en los siguiente valores: Kg_Pmax_Pre = 90,8077.

Análisis de varianza univariante con convariable *Pm_MaxP_Pos*

Factores inter-sujetos

		Etiqueta del valor	N
Grupo	1,00	Control	12
	2,00	Beta-alanina	14

Estadísticos descriptivos

Variable dependiente:***Pm_MaxP_Pos***

Grupo	Media	Desviación típica	N
Control	725,0833	106,84352	12
Beta-alanina	758,5000	96,33336	14
Total	743,0769	100,68324	26

Contraste de Levene sobre la igualdad de las varianzas error[a]

Variable dependiente:Pm_MaxP_Pos

F	gl1	gl2	Sig.
1,581	1	24	,221

Contrasta la hipótesis nula de que la varianza error de la variable dependiente es igual a lo largo de todos los grupos.
a. Diseño: Intersección + Pm_MaxP_Pre + Grupo

Pruebas de los efectos inter-sujetos

Variable dependiente:Pm_MaxP_Pos

Origen	Suma de cuadrados tipo III	gl	Media cuadrática	F	Sig.	Eta al cuadrado parcial	Parámetro de no centralidad Parámetro	Potencia observada[b]
Modelo corregido	176492,824[a]	2	88246,412	26,382	,000	,696	52,763	1,000
Intersección	55079,403	1	55079,403	16,466	,000	,417	16,466	,973
Pm_MaxP_Pre	169277,394	1	169277,394	50,606	,000	,688	50,606	1,000
Grupo	16368,647	1	16368,647	4,893	,037	,175	4,893	,563
Error	76935,023	23	3345,001					
Total	1,461E7	26						
Total corregida	253427,846	25						

a. R cuadrado = ,696 (R cuadrado corregida = ,670)
b. Calculado con alfa = ,05

Medias marginales estimadas

Grupo

Variable dependiente:Pm_MaxP_Pos

Grupo	Media	Error típ.	Intervalo de confianza 95%	
			Límite inferior	Límite superior
Control	715,823[a]	16,746	681,180	750,466
Beta-alanina	766,438[a]	15,498	734,378	798,497

a. Las covariables que aparecen en el modelo se evalúan en los siguiente valores: Pm_MaxP_Pre = 642,0769.

Análisis de varianza univariante con convariable *RM_kg_Pos*

Factores inter-sujetos

		Etiqueta del valor	N
Grupo	1,00	Control	12
	2,00	Beta-alanina	14

Estadísticos descriptivos

Variable dependiente:***RM_kg_Pos***

Grupo	Media	Desviación típica	N
Control	139,3333	15,12574	12
Beta-alanina	148,5000	17,73198	14
Total	144,2692	16,90812	26

Contraste de Levene sobre la igualdad de las varianzas error[a]

Variable dependiente:RM_kg_Pos

F	gl1	gl2	Sig.
5,734	1	24	,025

Contrasta la hipótesis nula de que la varianza error de la variable dependiente es igual a lo largo de todos los grupos.
a. Diseño: Intersección + RM_kg_Pre + Grupo

Pruebas de los efectos inter-sujetos

Variable dependiente:RM_kg_Pos

Origen	Suma de cuadrados tipo III	gl	Media cuadrática	F	Sig.	Eta al cuadrado parcial	Parámetro de no centralidad Parámetro	Potencia observada[b]
Modelo corregido	6003,795[a]	2	3001,897	60,389	,000	,840	120,777	1,000
Intersección	1264,442	1	1264,442	25,437	,000	,525	25,437	,998
RM_kg_Pre	5460,846	1	5460,846	109,855	,000	,827	109,855	1,000
Grupo	484,044	1	484,044	9,737	,005	,297	9,737	,848
Error	1143,320	23	49,710					
Total	548301,000	26						
Total corregida	7147,115	25						

a. R cuadrado = ,840 (R cuadrado corregida = ,826)
b. Calculado con alfa = ,05

Medias marginales estimadas

Grupo

Variable dependiente:RM_kg_Pos

Grupo	Media	Error típ.	Intervalo de confianza 95%	
			Límite inferior	Límite superior
Control	139,608[a]	2,035	135,397	143,819
Beta-alanina	148,265[a]	1,884	144,366	152,163

a. Las covariables que aparecen en el modelo se evalúan en los siguiente valores: RM_kg_Pre = 124,2692.

Análisis de varianza univariante con convariable *RM_Pm_Pos*

Factores inter-sujetos

		Etiqueta del valor	N
Grupo	1,00	Control	12
	2,00	Beta-alanina	14

Estadísticos descriptivos

Variable dependiente:RM_Pm_Pos

Grupo	Media	Desviación típica	N
Control	474,8000	104,57919	12
Beta-alanina	559,7050	112,19876	14
Total	520,5181	114,98461	26

Contraste de Levene sobre la igualdad de las varianzas error[a]

Variable dependiente:RM_Pm_Pos

F	gl1	gl2	Sig.
,027	1	24	,870

Contrasta la hipótesis nula de que la varianza error de la variable dependiente es igual a lo largo de todos los grupos.

a. Diseño: Intersección + RM_Pm_Pre + Grupo

Pruebas de los efectos inter-sujetos

Variable dependiente:RM_Pm_Pos

Origen	Suma de cuadrados tipo III	gl	Media cuadrática	F	Sig.	Eta al cuadrado parcial	Parámetro de no centralidad Parámetro	Potencia observada[b]
Modelo corregido	102470,929[a]	2	51235,465	5,167	,014	,310	10,334	,773
Intersección	85282,883	1	85282,883	8,601	,007	,272	8,601	,802
RM_Pm_Pre	55890,610	1	55890,610	5,636	,026	,197	5,636	,623
Grupo	44686,011	1	44686,011	4,507	,045	,164	4,507	,529
Error	228065,579	23	9915,895					
Total	7374952,287	26						
Total corregida	330536,508	25						

a. R cuadrado = ,310 (R cuadrado corregida = ,250)

b. Calculado con alfa = ,05

Grupo

Variable dependiente:RM_Pm_Pos

Grupo	Media	Error típ.	Intervalo de confianza 95%	
			Límite inferior	Límite superior
Control	475,732[a]	28,749	416,261	535,202
Beta-alanina	558,907[a]	26,616	503,848	613,965

a. Las covariables que aparecen en el modelo se evalúan en los siguiente valores: RM_Pm_Pre = 393,7692.

Análisis de varianza univariante con convariable *RM_Pp_Pos*

Factores inter-sujetos

		Etiqueta del valor	N
Grupo	1,00	Control	12
	2,00	Beta-alanina	14

Estadísticos descriptivos

Variable dependiente:RM_Pp_Pos

Grupo	Media	Desviación típica	N
Control	1467,4167	334,48263	12
Beta-alanina	1599,6429	235,48856	14
Total	1538,6154	287,37085	26

Contraste de Levene sobre la igualdad de las varianzas error[a]

Variable dependiente:RM_Pp_Pos

F	gl1	gl2	Sig.
,337	1	24	,567

Contrasta la hipótesis nula de que la varianza error de la variable dependiente es igual a lo largo de todos los grupos.

a. Diseño: Intersección + RM_Pp_Pre + Grupo

Pruebas de los efectos inter-sujetos

Variable dependiente:RM_Pp_Pos

Origen	Suma de cuadrados tipo III	gl	Media cuadrática	F	Sig.	Eta al cuadrado parcial	Parámetro de no centralidad Parámetro	Potencia observada[b]
Modelo corregido	913283,040[a]	2	456641,520	9,123	,001	,442	18,246	,956
Intersección	1807873,022	1	1807873,022	36,118	,000	,611	36,118	1,000
RM_Pp_Pre	800311,017	1	800311,017	15,989	,001	,410	15,989	,969
Grupo	43842,724	1	43842,724	,876	,359	,037	,876	,146
Error	1151267,114	23	50055,092					
Total	6,362E7	26						
Total corregida	2064550,154	25						

a. R cuadrado = ,442 (R cuadrado corregida = ,394)

b. Calculado con alfa = ,05

Grupo

Variable dependiente:RM_Pp_Pos

Grupo	Media	Error típ.	Intervalo de confianza 95%	
			Límite inferior	Límite superior
Control	1493,832[a]	64,922	1359,530	1628,134
Beta-alanina	1577,001[a]	60,062	1452,753	1701,248

a. Las covariables que aparecen en el modelo se evalúan en los siguiente valores: RM_Pp_Pre = 1212,9615.

Análisis de varianza univariante con convariable *Media_Pm_Pos*

Factores inter-sujetos

		Etiqueta del valor	N
Grupo	1,00	Control	12
	2,00	Beta-alanina	14

Estadísticos descriptivos

Variable dependiente:Media_Pm_Pos

Grupo	Media	Desviación típica	N
Control	589,0700	73,09566	12
Beta-alanina	612,4407	79,91885	14
Total	601,6542	76,24520	26

Contraste de Levene sobre la igualdad de las varianzas error[a]

Variable dependiente:Media_Pm_Pos

F	gl1	gl2	Sig.
5,189	1	24	,032

Contrasta la hipótesis nula de que la varianza error de la variable dependiente es igual a lo largo de todos los grupos.

a. Diseño: Intersección + Media_Pm_Pre + Grupo

Pruebas de los efectos inter-sujetos

Variable dependiente:Media_Pm_Pos

Origen	Suma de cuadrados tipo III	gl	Media cuadrática	F	Sig.	Eta al cuadrado parcial	Parámetro de no centralidad Parámetro	Potencia observada[b]
Modelo corregido	102176,715[a]	2	51088,357	27,227	,000	,703	54,454	1,000
Intersección	23358,237	1	23358,237	12,449	,002	,351	12,449	,922
Media_Pm_Pre	98647,485	1	98647,485	52,574	,000	,696	52,574	1,000
Grupo	1989,550	1	1989,550	1,060	,314	,044	1,060	,167
Error	43156,540	23	1876,371					
Total	9557016,404	26						
Total corregida	145333,255	25						

a. R cuadrado = ,703 (R cuadrado corregida = ,677)

b. Calculado con alfa = ,05

Medias marginales estimadas

Grupo

Variable dependiente:Media_Pm_Pos

Grupo	Media	Error típ.	Intervalo de confianza 95%	
			Límite inferior	Límite superior
Control	592,195[a]	12,512	566,312	618,078
Beta-alanina	609,762[a]	11,583	585,801	633,723

a. Las covariables que aparecen en el modelo se evalúan en los siguiente valores: Media_Pm_Pre = 510,7362.
