# Supplementary material for: Effects of β-alanine supplementation during a 5-week strength training program: a randomized, controlled study
Source: J Int Soc Sports Nutr. 2018 Apr 25;15:19. doi: 10.1186/s12970-018-0224-0 (PMC5918575; doi:10.1186/s12970-018-0224-0)
Supplement: Supplementary file 3 — Univariate general linear model. (PDF 169 kb) [file 12970_2018_224_MOESM3_ESM.pdf]

ADDITIONAL FILE 3

Análisis de varianza univariante- SERIES

Factores inter-sujetos

		Etiqueta del valor	N
grupos_Placebo_BA_Series	1,00	placeb	12
	2,00	Betaalanina	14

Estadísticos descriptivos

Variable dependiente:Post_Pre_Placebo_BA_Series

grupos_Placebo_BA_Series	Media	Desviación típica	N
placeb	1,5833	1,44338	12
Betaalanina	2,7857	1,12171	14
Total	2,2308	1,39449	26

Contraste de Levene sobre la igualdad de las varianzas error[a]

Variable dependiente:Post_Pre_Placebo_BA_Series

F	gl1	gl2	Sig.
2,404	1	24	,134

Contrasta la hipótesis nula de que la varianza error de la variable dependiente es igual a lo largo de todos los grupos.
a. Diseño: Intersección + grupos_Placebo_BA_Series

Pruebas de los efectos inter-sujetos

Variable dependiente:Post_Pre_Placebo_BA_Series

Origen	Suma de cuadrados tipo III	gl	Media cuadrática	F	Sig.	Eta al cuadrado parcial	Parámetro de no centralidad Parámetro	Potencia observada[b]
Modelo corregido	9,342[a]	1	9,342	5,709	,025	,192	5,709	,630
Intersección	123,342	1	123,342	75,373	,000	,758	75,373	1,000
grupos_Placebo_BA_Series	9,342	1	9,342	5,709	,025	,192	5,709	,630
Error	39,274	24	1,636					
Total	178,000	26						
Total corregida	48,615	25						

a. R cuadrado = ,192 (R cuadrado corregida = ,158)

b. Calculado con alfa = ,05

Medias marginales estimadas

grupos_Placebo_BA_Series

Estimaciones

Variable dependiente:Post_Pre_Placebo_BA_Series

grupos_Placebo_BA_Series	Media	Error típ.	Intervalo de confianza 95%	
			Límite inferior	Límite superior
placeb	1,583	,369	,821	2,345
Betaalanina	2,786	,342	2,080	3,491

Comparaciones por pares

Variable dependiente:Post_Pre_Placebo_BA_Series

(I)grupos_Placebo_BA_Series	(J)grupos_Placebo_BA_Series	Diferencia de medias (I-J)	Error típ.	Sig.[a]	Intervalo de confianza al 95 % para la diferencia[a]	
					Límite inferior	Límite superior
placeb	Betaalanina	-1,202*	,503	,025	-2,241	-,164
Betaalanina	placeb	1,202*	,503	,025	,164	2,241

Basadas en las medias marginales estimadas.

*. La diferencia de medias es significativa al nivel ,05.

a. Ajuste para comparaciones múltiples: Bonferroni.

Contrastes univariados

Variable dependiente:Post_Pre_Placebo_BA_Series

	Suma de cuadrados	gl	Media cuadrática	F	Sig.	Eta al cuadrado parcial	Parámetro de no centralidad Parámetro	Potencia observada[a]
Contraste	9,342	1	9,342	5,709	,025	,192	5,709	,630
Error	39,274	24	1,636					

Cada prueba F contrasta el efecto simple de grupos_Placebo_BA_Series en cada combinación de niveles del resto de los efectos mostrados.

a. Calculado con alfa = ,05

Análisis de varianza univariante- KILOGRAMS

Factores inter-sujetos

		Etiqueta del valor	N
grupos_Placebo_BA_KG	1,00	Placebo	12
	2,00	Betaalanina	14

Estadísticos descriptivos

Variable dependiente:Post_Pre_Placebo_BA_KG

grupos_Placebo_BA_KG	Media	Desviación típica	N
Placebo	15,4167	5,82250	12
Betaalanina	23,9286	9,64337	14
Total	20,0000	9,05539	26

Contraste de Levene sobre la igualdad de las varianzas error[a]

Variable dependiente:Post_Pre_Placebo_BA_KG

F	gl1	gl2	Sig.
2,352	1	24	,138

Contrasta la hipótesis nula de que la varianza error de la variable dependiente es igual a lo largo de todos los grupos.

a. Diseño: Intersección + grupos_Placebo_BA_KG

Pruebas de los efectos inter-sujetos

Variable dependiente:Post_Pre_Placebo_BA_KG

Origen	Suma de cuadrados tipo III	gl	Media cuadrática	F	Sig.	Eta al cuadrado parcial	Parámetro de no centralidad Parámetro	Potencia observada[b]
Modelo corregido	468,155[a]	1	468,155	7,103	,014	,228	7,103	,725
Intersección	10002,770	1	10002,770	151,764	,000	,863	151,764	1,000
grupos_Placebo_BA_KG	468,155	1	468,155	7,103	,014	,228	7,103	,725
Error	1581,845	24	65,910					
Total	12450,000	26						
Total corregida	2050,000	25						

a. R cuadrado = ,228 (R cuadrado corregida = ,196)

b. Calculado con alfa = ,05

Medias marginales estimadas
grupos_Placebo_BA_KG

Estimaciones

Variable dependiente:Post_Pre_Placebo_BA_KG

grupos_Placebo_BA_KG	Media	Error típ.	Intervalo de confianza 95%	
			Límite inferior	Límite superior
Placebo	15,417	2,344	10,580	20,254
Betaalanina	23,929	2,170	19,450	28,407

Comparaciones por pares

Variable dependiente:Post_Pre_Placebo_BA_KG

(I)grupos_Placebo_BA_KG	(J)grupos_Placebo_BA_KG	Diferencia de medias (I-J)	Error típ.	Sig.[a]	Intervalo de confianza al 95 % para la diferencia[a]	
					Límite inferior	Límite superior
Placebo	Betaalanina	-8,512*	3,194	,014	-15,104	-1,920
Betaalanina	Placebo	8,512*	3,194	,014	1,920	15,104

Basadas en las medias marginales estimadas.

*. La diferencia de medias es significativa al nivel ,05.

a. Ajuste para comparaciones múltiples: Bonferroni.

Contrastes univariados

Variable dependiente:Post_Pre_Placebo_BA_KG

	Suma de cuadrados	gl	Media cuadrática	F	Sig.	Eta al cuadrado parcial	Parámetro de no centralidad Parámetro	Potencia observada[a]
Contraste	468,155	1	468,155	7,103	,014	,228	7,103	,725
Error	1581,845	24	65,910					

Cada prueba F contrasta el efecto simple de grupos_Placebo_BA_KG en cada combinación de niveles del resto de los efectos mostrados.

a. Calculado con alfa = ,05
